# Influence of yoga on the epigenetics of obesity: a study on its short- and medium-term benefits in the treatment of patients with severe/morbid obesity eligible for bariatric surgery

**DOI:** 10.3389/fpubh.2026.1858629

**Published:** 2026-10-09

**Authors:** Carmela Asteria, Giorgia Scichilone, Rosanna Cardani, Dejan Lazarevic, Valentina Milani, Federico Ambrogi, Laura Valentina Renna, Marco Morelli, Francesca Giannese, Anna Sofia Tascini, Nicoletta Caridi, Fabiana Barbieri, Michela Ravelli, Alessandro Giovanelli

**Affiliations:** 1 Endocrine-Metabolic Nutrition Center, IRCCS, Ospedale Galeazzi-Sant’Ambrogio, Milan, Italy; 2 National Institute of Obesity Cure (INCO)-Bariatric Unit, IRCCS, Ospedale Galeazzi-Sant’Ambrogio, Milan, Italy; 3 BioCor Biobank, IRCCS Policlinico San Donato, Milan, Italy; 4 Center for Omics Sciences, IRCCS San Raffaele Scientific Institute, Vita-Salute San Raffaele University, Milan, Italy; 5 IRCCS Policlinico San Donato, Scientific Directorate, Milan, Italy; 6 Department of Clinical Sciences and Community Health, University of Milan, Milan, Italy

**Keywords:** bariatric surgery, epigenetics, inflammation, methylation, neuroendocrine, obesity, yoga

## Abstract

**Background:**

Obesity is a growing global health problem characterised by chronic low-grade inflammation and epigenetic dysregulation. Epigenetic mechanisms—potentially reversible changes in gene expression occurring without alteration of the DNA sequence, most notably DNA methylation—are increasingly implicated in obesity-related inflammation. Yoga may act as complementary therapy.

**Objective:**

To explore whether participation in a structured yoga program is associated with changes in DNA methylation of TNF-α, and with clinical, hormonal, metabolic and psychological parameters, in patients with severe obesity awaiting bariatric surgery.

**Methods:**

In this prospective, non-randomised, controlled observational cohort study, 40 patients were analysed (20 yoga, 20 control). The intervention group underwent 36 PRANA_it yoga sessions over the 3 months preceding surgery (mean attendance 26/36, 72.5%). DNA methylation was profiled using a targeted three-gene inflammatory panel (TNF-α, IL-6, CRP). Between-group differences were estimated by baseline-adjusted ANCOVA.

**Results:**

Plasma noradrenaline was significantly lower in the yoga group at T1 after adjustment for baseline [adjusted difference −206.92, 95% CI (−349.62, −64.21), *p* = 0.0057]. SF-36 physical functioning differed between groups in the unadjusted change-score comparison (*p* = 0.0206) but not after baseline adjustment [−8.69, 95% CI (−21.74, 4.36), *p* = 0.185]. Circulating TNF-α protein changed significantly more in the yoga group in the unadjusted analysis (*p* = 0.0452), a finding with high variance. Vitamin D3 showed no significant adjusted difference at T1 (*p* = 0.879); the T2 difference was borderline (*p* = 0.064). DNA methylation of TNF-α, IL-6 and CRP showed no significant changes after correction for multiple comparisons. Psychological outcomes, a secondary domain, did not differ significantly between groups.

**Conclusion:**

In patients with severe obesity preparing for bariatric surgery, a structured preoperative yoga program was associated with a significant baseline-adjusted reduction in plasma noradrenaline, consistent with reduced sympathetic activation during a demanding phase of care. No epigenetic changes were detected, suggesting that measurable methylation remodelling may require longer exposure, larger samples, or tissue-specific sampling. These exploratory findings support yoga as a feasible, low-risk adjunct within bariatric prehabilitation, while the non-randomised design precludes causal inference. Adequately powered randomised trials with extended follow-up are needed to confirm this neuroendocrine signal.

## Highlights

- The unadjusted change-score comparison in SF-36 physical functioning between T0 and T1 was nominally significant (*p* = 0.0206); however, the baseline-adjusted ANCOVA was not significant (*p* = 0.185).
- Plasma noradrenaline was significantly lower in the yoga group at T1 in the baseline-adjusted ANCOVA [adjusted difference = −206.92; 95% CI (−349.62, −64.21); *p* = 0.0057], consistent with reduced sympathetic activation.
- Circulating TNF-α protein showed a significant between-group difference at T0→T1 (*p* = 0.0452, high variance); no significant change was seen in TNF-α, IL-6, or CRP DNA methylation.
- Vitamin D3 showed no significant adjusted between-group difference at T1 (*p* = 0.879); the adjusted T2 difference was borderline (*p* = 0.064).
- Psychological outcomes were assessed as a secondary domain and did not differ significantly between groups.
- This is among the first studies to integrate epigenetic, clinical, hormonal, and psychological outcomes of yoga specific
- Ally in patients with severe/morbid obesity undergoing bariatric-surgery preparation.

## 1 Introduction

Over the past four decades, the global prevalence of obesity has nearly tripled. Recent estimates indicate that nearly 2 billion adults are overweight, with over 600 million classified as obese. Alarmingly, more than 40 million children under the age of five are also affected. Once perceived as a problem of industrialized nations, obesity is now rapidly rising in low-and middle-income countries, particularly in urbanized areas, where the rate of increase in childhood obesity has surpassed that of developed nations by more than 30%. As a result, the World Health Organization has declared obesity a global pandemic (1).

Obesity is a chronic disease and a major risk factor for several non-communicable diseases (NCDs), including metabolic syndrome, cardiovascular disease, musculoskeletal disorders such as osteoarthritis, venous stasis, thromboembolic events, chronic kidney disease, non-alcoholic steatohepatitis (NASH), and polycystic ovary syndrome (PCOS). It also increases the risk of several cancers, including those of the colon, liver, gallbladder, breast, endometrium, ovary, prostate, and kidney (2, 3).

The pathophysiology of metabolic syndrome—frequently co-occurring with obesity—is closely linked to sedentary lifestyles, high perceived stress, and poor diet. While diagnostic criteria vary across definitions, nearly all include abdominal obesity, hypertension, hyperglycemia, and dyslipidemia. Central to this syndrome are insulin resistance and adipose tissue dysfunction, which mediate systemic inflammation and cardiovascular risk (4, 5).

Obesity and its comorbidities are responsible for approximately one in five deaths globally and account for roughly 7% of healthcare expenditures across the European Union; indirect costs, such as work absenteeism and reduced productivity due to poor health and premature mortality, further exacerbate its economic burden (6).

### 1.1 Lifestyle-based and surgical approaches

Management of obesity requires multilevel therapeutic strategies, beginning with lifestyle interventions aimed at modifying diet and physical activity. Pharmacological treatment is considered only when lifestyle modification fails, while bariatric surgery is recommended in cases of severe or morbid obesity [Body Mass Index (BMI) ≥ 40 kg/m^2^ or ≥ 35 kg/m^2^ with comorbidities] (7). Among lifestyle-based therapies, yoga has emerged as a holistic, low-cost, and non-invasive intervention with promising benefits across multiple systems.

### 1.2 The role of yoga in obesity care

Yoga originated over 5,000 years ago in India as a spiritual practice rooted in Vedic philosophy, which underpins Hinduism, Buddhism, and Tantra (8). The word “Yoga” derives from the Sanskrit root yuj, meaning “to unite”—reflecting the practice’s aim to harmonize body, mind, and spirit.

Kundalini Yoga, in particular, is considered a potent form that works through specific sequences of movement, breathing, mantra, and meditation, and is often referred to as a fast-track form, enabling practitioners to achieve awareness and balance more quickly than traditional Hatha yoga (8, 9).

Yoga’s potential health benefits have been extensively documented, with evidence supporting improvements in metabolic function (lipid and glucose profiles) (10); cardiovascular health, including blood pressure and heart-rate modulation (11–14); endocrine regulation, such as thyroid function and vitamin D levels (15, 16); neuroendocrine and autonomic balance, with reductions in cortisol and adrenaline and increases in melatonin, endorphins, and GABA (17, 18); inflammation, with lowered IL-6, CRP, and TNF-α and increased adiponectin (19); immune function, with enhanced CD4 + cells, helper T-cells, and telomerase activity (20); and mental health, with reduced anxiety, depression, and stress and enhanced quality of life (21, 22).

Of particular scientific interest is the hypothesis that yoga may exert its effects at the molecular level, through epigenetic modulation of inflammatory genes.

### 1.3 Yoga and epigenetics: a new frontier

Epigenetics refers to heritable and potentially reversible changes in gene expression that occur without alterations to the underlying DNA sequence. The best-characterized epigenetic mechanism, DNA methylation, involves the addition of methyl groups to cytosine bases (typically at CpG dinucleotides), which can silence or activate gene transcription depending on genomic location; other mechanisms include histone modification and non-coding RNA regulation. In obesity, chronic low-grade inflammation is associated with hypomethylation of key inflammatory genes, potentially increasing expression of cytokines such as TNF-α, IL-6, and CRP. Evidence from a small pilot study involving 28 healthy women under chronic stress showed decreased methylation of TNF-α following yoga, suggesting enhanced gene expression and immune modulation (23).

To our knowledge, no previous study has evaluated the epigenetic effects of yoga specifically in patients with severe or morbid obesity, nor combined epigenetic, clinical, hormonal, and psychological outcomes within a single pre-bariatric-surgery protocol. This study addresses that gap: can yoga alter the epigenetic landscape in patients already experiencing metabolic and inflammatory dysregulation, such as those with obesity?

This study examines yoga as a complementary intervention in the preoperative management of severe obesity, with emphasis on neuroendocrine, inflammatory, and epigenetic outcomes; psychological well-being is assessed as a secondary domain.

## 2 Objectives

The overarching objective of this study is to explore the association between regular yoga practice—over a three-month period prior to bariatric surgery—and the epigenetic, metabolic, hormonal, inflammatory, and psychological profile of individuals with severe obesity. In particular, this research aims to determine whether the integration of yoga as a complementary intervention in the pre-surgical management of obesity is associated with clinically and molecularly measurable differences, especially at the level of gene methylation in key inflammatory pathways. Given the non-randomised, observational design, these objectives are framed as exploratory and associative rather than causal.

### 2.1 Primary objective

To assess whether regular yoga practice is associated with a measurable difference, between T0 and T1, in mean DNA methylation (percentage 5-methylcytosine) across the promoter/transcription-start-site region of TNF-α (chr6:31,574,365–31,578,336, GRCh38/hg38), compared between the yoga and control groups.

### 2.2 Secondary objectives

To assess the association between yoga and molecular, physiological, and psychological parameters across the domains summarized in Table 1.

**Table 1:** Secondary objectives.

Domain	Parameters assessed
Epigenetic modulation of additional inflammatory biomarkers	Interleukin-6 (IL-6); C-reactive protein (CRP)
Circulating levels of inflammatory and metabolic biomarkers	TNF-α, IL-6, CRP; leptin, adiponectin; β-endorphin, melatonin, GABA
Anthropometric changes	Body weight; visceral fat mass; waist-to-hip ratio; BMI
Metabolic improvements	Insulin resistance (HOMA-IR); HbA1c, fasting glucose, OGTT; lipid profile
Hormonal and endocrine status	TSH, ACTH, cortisol; vitamin D3; thyroid antibodies (AbTPO)
Neuroendocrine markers	Plasma noradrenaline; salivary cortisol; catecholamines
Immune function parameters	CD4 + lymphocytes, ANA; T-helper cells
Psychological outcomes	Health-related quality of life (SF-36); state anxiety (STAI-Y); depressive symptoms (BDI-II)

### 2.3 Study hypothesis

We hypothesize that integrating yoga into the preoperative routine of bariatric candidates is associated with short-and medium-term clinical differences, and possibly molecular changes, through modulation of inflammatory pathways and neuroimmune homeostasis, potentially reflected in reduced stress and anxiety, improved mood and quality of life, enhanced metabolic control, reduced inflammatory burden, and altered methylation patterns in key inflammatory genes.

### 2.4 Time points for assessment

All parameters were assessed at three standardized time points: T0 (baseline, at enrollment); T1 (pre-hospitalization, after 3 months of yoga practice or observation in controls, and before surgery); and T2 (3 months post-surgery, to assess intermediate postoperative effects). Comparisons were made between groups (Yoga vs. Control) and within groups over time (T1 vs. T0; T2 vs. T0).

## 3 Materials and methods

### 3.1 Study design

This was a monocentric, prospective, non-randomized, controlled observational cohort study (yoga vs. standard-care comparison group) conducted at IRCCS Policlinico San Donato (Milan, Italy). No randomisation or blinding of group allocation was performed. The study enrolled 48 patients aged between 18 and 65 years, all with severe or morbid obesity and eligible for bariatric surgery. Participants were allocated into two groups: a yoga group (*n* = 20), which received a structured yoga intervention for 3 months prior to surgery, and a control group (*n* = 20), which received standard care with no structured physical or psychological intervention. DNA methylation was profiled using a targeted three-gene inflammatory panel (TNF-α, IL-6, CRP; promoter and transcription-start-site regions; see Section 3.6 for genomic coordinates and sequencing methodology). Each patient underwent evaluations at three timepoints: T0 (baseline), T1 (pre-hospitalization, after the yoga or observation period), and T2 (3 months post-surgery).

### 3.2 Inclusion and exclusion criteria

Eligible participants were adults with a BMI ≥ 35 kg/m^2^ with obesity-related comorbidities or a BMI ≥ 40 kg/m^2^, who met national eligibility criteria for bariatric surgery, had a diagnosis of metabolic syndrome according to ATP IV criteria, and provided signed informed consent. Patients were excluded if they had undergone prior bariatric procedures, had uncontrolled endocrinological or psychiatric disorders, were pregnant, had active substance abuse, or had any condition preventing yoga practice or long-term follow-up.

### 3.3 Recruitment and allocation

Patients referred for bariatric-surgery evaluation at IRCCS Policlinico San Donato (Milan, Italy) between February and October 2022 were screened consecutively against the inclusion and exclusion criteria above. Eligible patients were informed about the study and provided written informed consent. Participation in the yoga program was offered as an optional component of routine preoperative care; patients entered the yoga or the control group according to their own availability and willingness to attend the scheduled sessions, and investigators did not randomize, assign, or allocate participants to either group. The study was therefore conducted and is reported as an observational cohort study rather than as a clinical trial, and prospective registration in a clinical trial registry was accordingly not required under ICMJE criteria for this design. Of 48 patients initially enrolled, 40 (20 per group) completed baseline assessment and at least one follow-up visit and were included in the analysis (see Section 4, Figure 1, for the full participant flow and reasons for exclusion).

**Figure 1:**
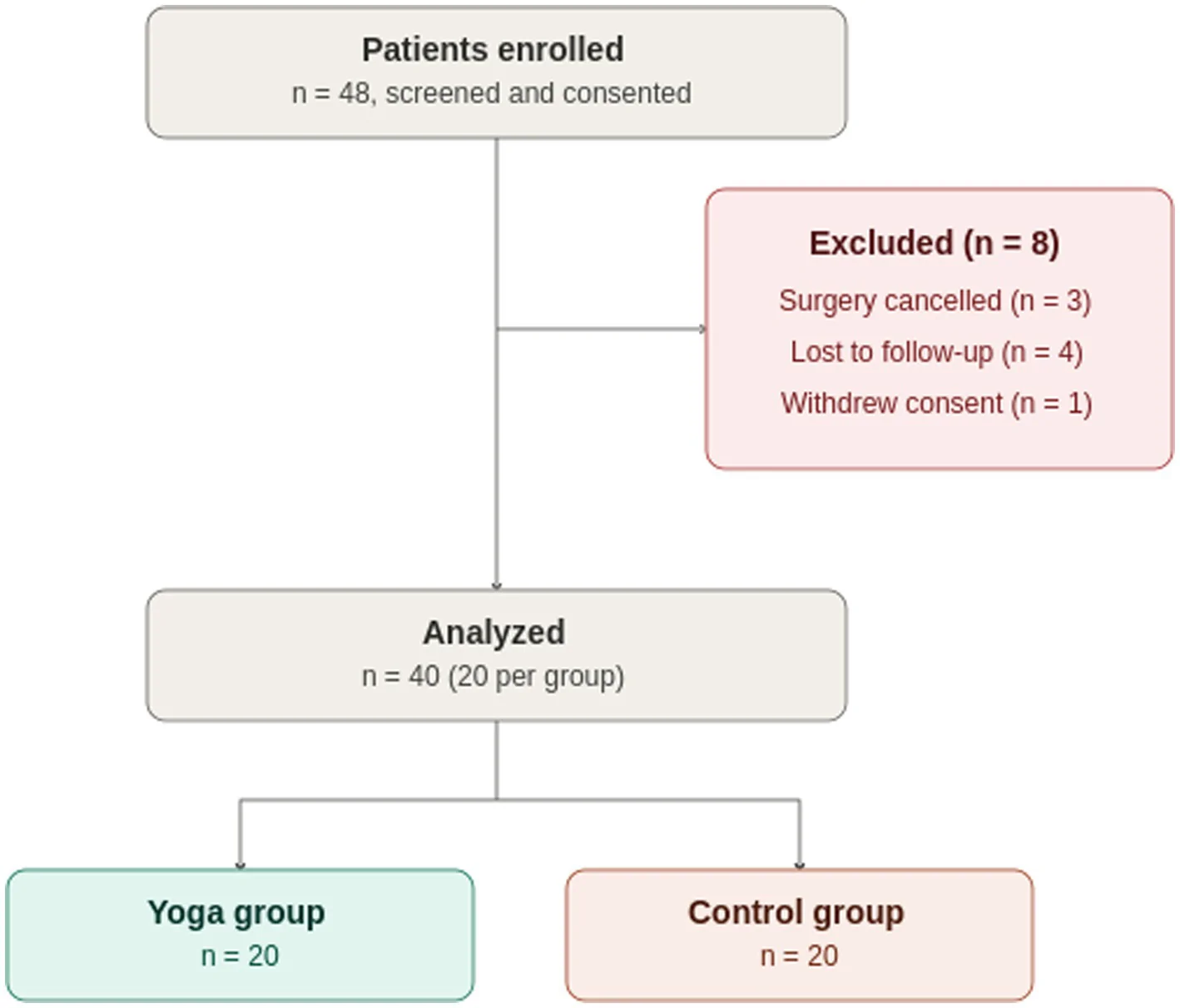
CONSORT-style participant flow diagram. Of the 8 patients excluded between enrollment and analysis: 3 had surgery cancelled, 4 were lost to follow-up, and 1 withdrew consent.

### 3.4 Yoga intervention: PRANA_it protocol

The intervention arm received 36 supervised group sessions of the PRANA_it yoga protocol, delivered at a frequency of approximately three sessions per week over a 3-month period, each lasting 45–60 min. Sessions were led by instructors certified in the PRANA_it method, holding a 300-h Kriya Yoga certification (Kriya Yoga Center, Via degli Olivetani, Milan, Italy), a 300-h first-level Kundalini Yoga certification, and registration with IKYTA Italia (International Kundalini Yoga Teachers’ Association) as a teacher member at the third level, including completed specializations; the lead instructor was, at the time of writing, undertaking a fourth-level international Kriya Yoga training program in collaboration with instructors in California, United States, and had completed Karam Kriya training with Shiv Charan Singh in Portugal. All the sessions were structured in three sequential phases: a warm-up phase emphasizing breath awareness, core activation, and spinal alignment; a kriya phase comprising Kundalini-inspired sequences of movement, pranayama (breathing techniques), and mantra; and a closing breathing-and-relaxation phase to promote parasympathetic activation. Intensity was low-to-moderate and adapted to individual mobility limitations associated with severe obesity, with modifications offered for seated or supported postures as needed. Across the 36 sessions, patients showed a progressive improvement in their ability to reduce reliance on seated or supported modifications as tolerated. Session attendance was recorded by instructors at each class; participants attended a mean of 26/36 sessions (72.5%). Adherence to any additional home-based practice was not systematically monitored, which is acknowledged as a limitation (Section 5.1).

### 3.5 Sample collection and biobanking

At each timepoint, whole blood and serum samples were collected and processed at the BioCor Biobank of IRCCS Policlinico San Donato following standardized standard operating procedures. Whole blood was collected in EDTA tubes (7.2 mg K3), aliquoted into 2D-barcoded cryotubes, and stored at −80 °C. Serum was collected in clot-activator tubes, allowed to coagulate for 30 min, centrifuged at 1500 RCF, manually separated, and cryopreserved. Sample tracking, registration, retrieval, and inventory control were managed using EasyTrack2 software.

### 3.6 DNA extraction and methylation profiling

Genomic DNA was extracted using the Maxwell RSC Blood DNA Kit (Promega) and quantified using a Qubit fluorometer (Thermo Fisher) and an Agilent TapeStation 4,200 for DNA integrity assessment (DNA Integrity Number > 7 required). A total of 129 samples were processed using the NEBNext® Enzymatic Methyl-seq Kit in conjunction with the Twist Targeted Methylation Protocol, avoiding bisulfite treatment to preserve DNA quality. Target regions were designed using the GRCh38/hg38 assembly for three genes: TNF-α (chr6:31,574,365–31,578,336; −1,200 bp from the transcription start site), IL-6 (chr7:22,725,884–22,732,002; −2000 bp from the transcription start site), and CRP (chr1:159,712,289–159,715,589; +2000 bp from the transcription start site, reverse strand). Libraries were sequenced on an Illumina NovaSeq 6,000 platform (100 bp paired-end), targeting approximately 10 million reads per sample. Reads were processed with bcl2fastq, trimmed with TrimGalore, mapped with Bismark, and analyzed using edgeR and methylKit. CpG sites were filtered for ≥8 × coverage, and unsupervised clustering was performed; no significant methylation differences emerged after correction for multiple testing.

### 3.7 Serum biomarker quantification (ELISA)

Serum levels of inflammatory, neuroendocrine, and metabolic markers were measured using manual ELISA kits at all time points (Table 2), with readings taken at 450 nm following standard-curve calibration.

**Table 2:** ELISA kits.

Analyte	ELISA kit	Dilution
IL-6	LSBio	1:10
Adiponectin	LSBio	1:2000
Melatonin	MyBioSource	Undiluted
TNF-α	MyBioSource	Undiluted
β-Endorphin	MyBioSource	Undiluted
Leptin	MyBioSource	1:100
GABA	MyBioSource	1:10

### 3.8 Psychological and quality-of-life assessments

Participants completed the SF-36 questionnaire to assess health-related quality of life (total and domain-specific scores), the Beck Depression Inventory (BDI-II) to assess depressive symptoms, and the state component of the State–Trait Anxiety Inventory (STAI-Y) to assess state anxiety.

### 3.9 Additional clinical and instrumental assessments

Anthropometric assessment included BMI, waist-to-hip ratio, and visceral fat mass measured by bioimpedance (seca mBCA 515). Metabolic markers assessed included glucose, HbA1c, insulin, lipid profile, and oral glucose tolerance testing (OGTT). The hormonal profile included TSH, FT3, FT4, cortisol, ACTH, vitamin D3, and thyroid antibodies. Neuroendocrine assessment comprised plasma noradrenaline and salivary cortisol. Immunological testing included CD4 count, antinuclear antibodies (ANA), and a T-helper panel. Hepatic ultrasound and electrocardiography were performed at T0 only.

### 3.10 Statistical analysis

Sample size was calculated based on anticipated changes in TNF-α methylation (estimated yoga-group mean = 0.332 ± 0.046 vs. control = 0.367 ± 0.048; α = 0.05; power = 80%). Normality of distributions was assessed using the Shapiro–Wilk test. Baseline between-group comparisons were performed using the independent-samples t-test for continuous variables (Table 3) and the chi-square test for categorical variables. Between-group comparisons of change scores (Δ = post-value—baseline) were performed using the Mann–Whitney U test (equivalent to the Wilcoxon rank-sum test for two independent samples), selected because Shapiro–Wilk testing indicated non-normal distributions for the majority of change scores; a standardized mean difference (Cohen’s d, calculated from group means and pooled SD of the change scores) is reported alongside each *p*-value as a descriptive effect-size estimate. Time-course evaluation used mixed-effects models and repeated-measures ANOVA. Differential DNA methylation was tested using edgeR and methylKit, with false discovery rate (FDR) correction for multiple testing. The significance threshold was set at *p* < 0.05 unless otherwise specified. Full statistical tables for all 46 assessed variables at both T0 → T1 and T0 → T2 are provided in Supplementary Tables S2, S3; see the Supplement for further methodological detail. For the primary preoperative comparisons, between-group differences were estimated using analysis of covariance (ANCOVA), modeling the T1 (or T2) value as a function of the T0 baseline value and group assignment, yielding an adjusted between-group difference with 95% confidence interval. This baseline-adjusted model is reported as the primary confirmatory analysis; the unadjusted Mann–Whitney U comparison of raw change scores (Section 3.10, original approach) is retained as a secondary, descriptive analysis. Results of this approach are reported in Table 4.

**Table 3:** Baseline sociodemographic, anthropometric, and clinical characteristics by group.

Variable	Control (*n* = 20) mean ± SD	Yoga (*n* = 20) mean ± SD	*t*(df = 38)	*p*-value	Cohen’s *d*
Sociodemographics					
Age (years)	46.03 ± 13.07	47.11 ± 12.46	−0.27	0.7886	−0.085
Anthropometrics					
Height (m)	1.66 ± 0.10	1.61 ± 0.10	1.54	0.1318	0.487
Weight (kg)	114.91 ± 18.31	114.60 ± 19.85	0.05	0.9604	0.016
BMI (kg/m^2^)	41.83 ± 5.08	43.83 ± 5.66	−1.18	0.2453	−0.373
Waist circumference (cm)	112.60 ± 10.44	114.30 ± 12.59	−0.46	0.6481	−0.145
Hip circumference (cm)	125.30 ± 9.78	129.05 ± 12.09	−1.08	0.2869	−0.342
Waist-to-hip ratio	0.90 ± 0.07	0.89 ± 0.09	0.41	0.6841	0.130
Psychological domains					
SF-36 physical functioning	55.75 ± 28.94	69.75 ± 21.97	−1.72	0.0936	−0.544
SF-36 general health	45.00 ± 19.60	55.00 ± 12.88	−1.91	0.0637	−0.604
STAI-1 state anxiety	46.20 ± 10.90	43.85 ± 8.30	0.77	0.4461	0.243
STAI-2 trait anxiety	46.15 ± 10.80	41.40 ± 10.07	1.44	0.1581	0.455
BDI depression	11.10 ± 5.47	11.00 ± 6.83	0.05	0.9604	0.016

Independent-samples *t*-test, df = 38. Cohen’s d = t × √(1/n1 + 1/n2). Sample sizes differ slightly from *n* = 48 originally enrolled because 8 participants (Figure 1) did not complete a baseline visit or were excluded before assessment.

## 4 Results

Forty-eight patients were screened and enrolled; forty completed baseline and at least one follow-up assessment and were included in the analysis (Yoga, *n* = 20; Control, *n* = 20). Table 3 summarizes the sociodemographic, anthropometric, and baseline primary-outcome characteristics of the two groups. Group comparisons at baseline were performed using the independent-samples t-test (two-tailed, df = 38); no statistically significant between-group differences were observed for any baseline variable (all *p* > 0.05), confirming adequate comparability between groups at enrollment (Figure 1).

### 4.1 Quality of life and psychological well-being

In the secondary, unadjusted change-score analysis, a statistically significant between-group difference emerged in the SF-36 physical functioning domain between T0 and T1 (Mann–Whitney U, *p* = 0.0206; Control +5.25 ± 28.63 vs. Yoga −14.00 ± 26.09; Cohen’s d = −0.703): the control group’s score improved on average, while the yoga group’s score declined. In the primary baseline-adjusted ANCOVA, however, the between-group difference at T1 was not statistically significant (n = 40; adjusted difference [Yoga—Control] = −8.69, SE = 6.44, 95% CI [−21.74, 4.36], *t*(37) = −1.35, *p* = 0.185).

Other SF-36 subdomains, such as role limitations due to physical health (Control +4.25 ± 35.44 vs. Yoga +13.75 ± 54.70, *p* = 0.2723), showed positive trends in the intervention group but did not reach statistical significance after accounting for multiple comparisons.

In terms of psychological health, depressive symptoms (BDI-II) decreased in both groups between T0 and T1 (Control −1.50 ± 1.76 vs. Yoga −4.15 ± 5.47), a larger numerical decrease in the yoga group that did not reach statistical significance (Mann–Whitney U, *p* = 0.1246; Cohen’s d = −0.652). State anxiety (STAI-Y1) also did not differ significantly between groups at T1, suggesting that the yoga intervention had a modest but not statistically conclusive impact on mood regulation during the short preoperative window.

### 4.2 Neuroendocrine and hormonal modulation

Yoga was associated with a significant reduction in plasma noradrenaline between T0 and T1 in the secondary, unadjusted change-score analysis (Mann–Whitney U, *p* = 0.0380; Control +147.60 ± 273.65 vs. Yoga −38.74 ± 149.83 pg./mL; Cohen’s d = −0.839), consistent with a shift in autonomic tone toward reduced sympathetic activation. The primary baseline-adjusted ANCOVA corroborated this finding (*n* = 39; adjusted difference [Yoga—Control] = −206.92, SE = 70.37, 95% CI [−349.62, −64.21], *t*(36) = −2.94, *p* = 0.0057).

In the secondary, unadjusted change-score analysis, serum vitamin D3 (25-OH D) concentrations rose by 14 ng/mL in the yoga group, nearly doubling the 6.4 ng/mL increase observed in controls (Mann–Whitney U, *p* = 0.0482). In the primary baseline-adjusted ANCOVA, the between-group difference at T1 was not statistically significant (*n* = 38; adjusted difference [Yoga—Control] = −0.54, SE = 3.52, 95% CI [−7.68, 6.60], *t*(35) = −0.15, *p* = 0.879); at T2, the adjusted difference was +11.94 (SE = 6.25, 95% CI [−0.75, 24.62], *t*(35) = 1.91, *p* = 0.064), indicating a borderline rather than statistically significant difference.

Serum cortisol and ACTH levels did not show significant changes between groups at T1, although trends toward normalization were observed postoperatively in both arms. Salivary cortisol measurements echoed these patterns.

### 4.3 Inflammatory and metabolic biomarkers

Circulating TNF-α, IL-6, and CRP were measured at each timepoint by ELISA. Between T0 and T1, circulating TNF-α changed significantly more in the yoga group than in controls (Mann–Whitney U, *p* = 0.0452; Control +2.73 ± 9.81 vs. Yoga +27.50 ± 195.74 pg./mL; Cohen’s d = 0.179).

IL-6 (Control −54.53 ± 132.84 vs. Yoga −41.88 ± 151.71 pg./mL, *p* = 0.9307) and CRP (Control +4.63 ± 27.57 vs. Yoga +0.43 ± 4.61 mg/L, *p* = 0.9889) did not differ significantly between groups between T0 and T1. Post-surgery, all participants showed substantial reductions in CRP, suggesting a robust anti-inflammatory response to weight loss, but no additional preoperative effect was attributable specifically to yoga.

Levels of adiponectin, a beneficial anti-inflammatory adipokine, showed a numerical increase in the yoga group, whereas leptin, typically elevated in obesity, remained largely unchanged across groups; these differences were not statistically significant.

Anthropometric measures were essentially unchanged between groups during the preoperative phase, as expected in patients awaiting bariatric surgery, and did not differ significantly at T2 either (BMI: Control −7.26 ± 5.58 vs. Yoga −8.37 ± 7.88 kg/m^2^, *p* = 0.7696; weight: Control −20.63 ± 18.12 vs. Yoga −20.64 ± 21.37 kg, *p* = 0.9444), indicating comparable surgical weight-loss trajectories in both arms. Fasting glucose, HbA1c, and insulin resistance (HOMA-IR) improved mildly in both groups without significant intergroup differences.

### 4.4 Epigenetic profiling

Targeted sequencing was conducted for TNF-α, IL-6, and CRP, focusing on their promoter regions and transcription start sites. High-quality methylation data were obtained from 129 samples, with an average read depth of approximately 10 million reads and stringent filtering for CpG site coverage.

Unsupervised clustering of methylation data across all time points revealed no clear segregation based on intervention (Yoga vs. Control) or on the timing of sample collection (T0–T2), suggesting that the overall epigenetic landscape remained relatively stable over the study period. Differential methylation analysis using edgeR and methylKit, corrected for multiple testing (FDR), did not identify any significantly altered CpG sites in either between-group or within-group comparisons. A limited number of CpG loci showed changes under less stringent, uncorrected thresholds (e.g., *p* < 0.05 and |logFC| > 1), particularly within TNF-α and IL-6 regions; these exploratory signals may inform future targeted studies with longer interventions or larger cohorts, but should not be interpreted as significant findings given the lack of correction.

### 4.5 Postoperative observations (T2)

Although not the primary focus of this paper, postoperative data at 3 months (T2) showed marked improvements in inflammatory markers, anthropometry, and metabolic control across both groups. These improvements were expected following bariatric surgery and were generally comparable between groups (Section 4.3), indicating that group differences observed during the preoperative phase did not persist, or were superseded by, the shared physiological effects of surgery.

### 4.6 Summary of Main findings

Table 5 summarizes the secondary, unadjusted between-group comparisons of change scores, while Table 4 reports the baseline-adjusted ANCOVA for selected outcomes. In the adjusted analysis, plasma noradrenaline showed a statistically significant between-group difference at T1, whereas SF-36 physical functioning and vitamin D3 did not; the vitamin D3 difference at T2 was borderline (*p* = 0.064). The unadjusted analysis also identified a nominally significant difference in circulating TNF-α protein at T0 → T1, with high variance. No significant differences in DNA methylation of TNF-α, IL-6, or CRP were detected. Full statistical results for all 46 assessed variables are provided in Supplementary Table S2 (T0 → T1) and S3 (T0 → T2).

**Table 4:** Baseline-adjusted ANCOVA results for selected outcomes.

Outcome/Time point (*n*)	Adjusted difference (Yoga—Control), SE	95% CI	*t*(df); *p*-value
SF-36 physical functioning, T1 (*n* = 40)	−8.69; SE 6.44	[−21.74, 4.36]	*t*(37) = −1.35; *p* = 0.185
Plasma noradrenaline, T1 (*n* = 39)	−206.92; SE 70.37	[−349.62, −64.21]	*t*(36) = −2.94; *p* = 0.0057
Vitamin D3, T1 (*n* = 38)	−0.54; SE 3.52	[−7.68, 6.60]	*t*(35) = −0.15; *p* = 0.879
Vitamin D3, T2 (*n* = 38)	+11.94; SE 6.25	[−0.75, 24.62]	*t*(35) = 1.91; *p* = 0.064

**Table 5:** Summary of unadjusted change-score findings.

Outcome	Direction	Yoga vs. Control	*p*-value
SF-36 physical functioning (Δ T0 → T1)	↓ in Yoga, ↑ in Control	Control improved; Yoga declined	0.0206
Plasma noradrenaline (Δ T0 → T1)	↓	Yoga reduced vs. Control increased	0.0380
TNF-α protein, ELISA (Δ T0 → T1)	↑	Larger increase in Yoga (high variance)	0.0452
Vitamin D3 (25-OH)	↑	Yoga increased (+14 vs. +6.4 ng/mL)	0.0482
DNA methylation (TNF-α, IL-6, CRP)	—	No significant difference (FDR-corrected)	>0.05 (FDR)
IL-6, CRP protein, ELISA (Δ T0 → T1)	—	No significant difference	>0.05

## 5 Discussion

This study explored the association between yoga, as a complementary intervention, and clinical, hormonal, and psychological parameters in patients with severe or morbid obesity awaiting bariatric surgery. Over a three-month period, the baseline-adjusted analysis supported a significant between-group difference in plasma noradrenaline, while SF-36 physical functioning and vitamin D3 were not significant after baseline adjustment at T1. The unadjusted analysis also identified a nominally significant between-group difference in circulating TNF-α protein, although this result had high variance and should be interpreted cautiously. These findings add to a growing body of evidence suggesting that yoga, beyond its well-known effects on flexibility and relaxation, may be associated with measurable biological differences relevant to metabolic and inflammatory conditions.

The unadjusted SF-36 physical-functioning result warrants exploratory comment, although it was not confirmed by the primary baseline-adjusted ANCOVA (*p* = 0.185). One plausible, literature-supported explanation is a response-shift phenomenon: as patients begin a new structured physical practice, they often become more attuned to pre-existing functional limitations that were previously outside their awareness, and may consequently recalibrate their internal standards for what constitutes “good” physical functioning—a change in the meaning of one’s self-evaluation of quality of life resulting from a shift in internal standards, values, or conceptualization, rather than a true change in the underlying health state (24). This phenomenon has been well documented in physical rehabilitation and exercise-intervention contexts, where patient-reported outcome measure (PROM) scores can paradoxically decline despite objective functional improvement. An alternative, non-exclusive explanation is that the control group’s improvement reflects a growing sense of optimism as the scheduled surgery date approached, independent of any yoga-specific effect. Distinguishing between these explanations was beyond the scope of the present descriptive comparison and represents an important direction for future research using appraisal-based or then-test methodologies designed to detect and adjust for response shift.

The observed reduction in noradrenaline levels is noteworthy, as it suggests a shift in autonomic balance toward parasympathetic predominance, associated with decreased sympathetic stress responses. This is broadly consistent with prior research showing that yoga can downregulate the hypothalamic–pituitary–adrenal (HPA) axis, reduce cortisol secretion, and modulate catecholamines, thereby improving resilience to stress (17–19).

The unadjusted increase in serum vitamin D3 observed in the yoga group should be considered exploratory, because the baseline-adjusted difference was not significant at T1 (*p* = 0.879) and was borderline at T2 (*p* = 0.064). While vitamin D status is influenced by many factors—including sun exposure, adiposity, and absorption efficiency—this result may reflect subtle changes in immune or endocrine function associated with yoga, as well as possibly increased outdoor exposure or enhanced bioavailability. Vitamin D is known to play roles in immune regulation, insulin sensitivity, and even epigenetic gene expression, making this change potentially meaningful in the context of metabolic health (16, 20).

The significant increase in circulating TNF-α protein in the yoga group between T0 and T1 (Section 4.3) is an important finding. Because it is accompanied by very high variance in the yoga arm, likely influenced by at least one extreme value, this result should be interpreted cautiously. However this result would suggest that yoga may be associated with changes in inflammatory protein expression independently of, or prior to, any detectable change in promoter methylation, and would represent an interesting point of divergence between the protein-level and epigenetic-level findings reported here.

Despite these clinical and endocrine findings, the study did not demonstrate significant changes in DNA methylation of the inflammatory genes TNF-α, IL-6, and CRP. While some exploratory signals emerged under relaxed, uncorrected statistical thresholds, these findings must be interpreted cautiously and require further validation. The absence of significant epigenetic changes may be attributable to several factors. First, the 3-month intervention window, while adequate to detect several clinical, hormonal, and psychological changes, may be insufficient to produce stable, detectable remodeling of DNA methylation in chronically dysregulated inflammatory loci; published epigenetic lifestyle-intervention studies more commonly employ 6–12-month exposures, and this is a key limitation of the present epigenetic findings (see Section 5.3). Second, peripheral blood, while accessible, may not fully capture tissue-specific methylation changes occurring in adipose, liver, or hypothalamic tissue. Third, the sample size, while adequate for detecting medium effect sizes in clinical parameters, may be underpowered for detecting subtler epigenetic differences.

Epigenetic regulation is also complex and context-dependent. While a previous pilot study in chronically stressed but otherwise healthy women reported changes in TNF-α methylation following yoga (23), our cohort included individuals with longstanding severe obesity and established inflammation, potentially reflecting a more entrenched epigenetic profile than that of healthy stressed adults. These results suggest that yoga’s molecular effects may be more readily detectable in earlier-stage or stress-related conditions than in advanced, chronic metabolic disease.

While TNF-α, IL-6, and CRP are clinically validated inflammatory biomarkers with established links to cardiometabolic risk, the clinical significance of peripheral-blood methylation changes at these loci—as opposed to circulating protein levels—has not yet been established in this population, and should be interpreted as an exploratory, mechanistic endpoint rather than a validated clinical marker. TNF-α, IL-6, and CRP were selected *a priori* as the primary epigenetic targets because they are central, well-validated mediators of obesity-related chronic inflammation, each is mechanistically linked to insulin resistance and cardiovascular risk, and TNF-α methylation was the only inflammatory-gene locus previously shown to be modulated by a brief yoga intervention (23), providing direct precedent for this targeted-panel replication design.

Interindividual variability in response to mind–body interventions is another important consideration. Factors such as genetic background, baseline stress levels, sleep patterns, and dietary habits could all influence the degree to which yoga is associated with biological changes. All participants, irrespective of group, received the center’s standard preoperative dietary and nutritional counselling as part of routine bariatric-surgery preparation. Preoperative body weight and BMI did not differ significantly between groups at T1, indicating that differential weight loss is unlikely to explain the observed clinical and hormonal differences. However, physical activity outside structured sessions and medication use were not systematically recorded or controlled between arms; this is acknowledged as a limitation, since these factors can independently influence both epigenetic and clinical outcomes and cannot be fully excluded as confounders in this non-randomised design.

Nonetheless, the absence of significant methylation differences does not negate the potential systemic associations of yoga observed in this study. The baseline-adjusted difference in autonomic tone is potentially meaningful in the context of surgical preparation and recovery, given that stress and inflammation are associated with worse postoperative outcomes, including longer hospital stays and impaired wound healing. As such, integrating yoga into the preoperative pathway may offer physiological value even in the absence of overt epigenetic change; any psychological benefit remains exploratory because the psychological secondary outcomes did not differ significantly between groups.

### 5.1 Limitations

This study has several limitations. The intervention period was short (3 months) relative to typical epigenome-remodeling timescales reported in lifestyle-intervention literature (commonly 6–12 months), and the entrenched, chronic inflammatory state of long-standing severe obesity likely requires longer exposure than the acute stress-reduction context of prior pilot work (23). The design was observational and non-randomised, with allocation based on patient availability rather than random assignment; while the groups were well matched at baseline (Table 3), the lack of randomisation introduces the potential for unmeasured confounders. Physical activity outside structured sessions, dietary intake beyond standard counselling, and medication use were not systematically recorded or controlled between arms and cannot be fully excluded as confounders. Adherence to the 36 supervised sessions was recorded (mean 26/36 sessions, 72.5%), but adherence to any additional home-based practice was not systematically monitored, introducing potential exposure variability within the intervention arm.

Because allocation was based on patients’ availability and willingness to attend sessions rather than random assignment, the yoga group may differ systematically from the control group in motivation, mobility, health literacy, adherence to medical advice, available time, socioeconomic circumstances, and psychological support, any of which could independently influence the outcomes studied. The control group was also not an attention-matched comparison; instructor contact, group participation, and associated expectancy or social-support effects cannot be distinguished from a yoga-specific physiological effect in the present design. These are fundamental limitations of the non-randomised, self-selected design that cannot be corrected retrospectively and should be weighed accordingly when interpreting all reported associations.

Gene expression of TNF-α was not assessed in this study and is not reported; this is a scope limitation, and future work should incorporate RT-qPCR or RNA-sequencing analysis to relate methylation changes to actual transcriptional output.

Adverse events were not systematically ascertained during the intervention period; the absence of a structured adverse-event monitoring procedure is acknowledged as a limitation of the reported safety profile.

Participants were recruited consecutively from a single tertiary bariatric-surgery referral center in an urban area of Northern Italy and are broadly representative of patients accessing bariatric care in this healthcare setting; however, as a single-center, non-randomised sample, generalizability to populations with different healthcare-access patterns, socioeconomic composition, or geographic and cultural context should be made cautiously.

Of 48 patients initially enrolled, 8 were excluded before analysis (3 due to surgery cancellation, 4 due to loss to follow-up, 1 due to withdrawal of consent, Figure 1), and effective sample sizes for individual biomarkers ranged from 12 to 20 per group owing to assay-level missing data (Supplementary Tables S2, S3), which should be considered when interpreting biomarker-specific results. Biochemical analyses were limited to a select panel of markers, and the study did not assess other potentially relevant pathways, such as mitochondrial function, telomere length, or transcriptomic shifts. The epigenetic analysis, while technically robust, was restricted to a narrow gene panel and a single tissue source (peripheral blood); a more comprehensive epigenomic or multi-omics approach may be better suited to capture the broader biological impact of mind–body practices.

### 5.2 Strengths

Despite these limitations, this study has several strengths. It is among the first to integrate epigenetic, clinical, endocrine, and psychological outcomes in the context of obesity and yoga, specifically in a population with severe/morbid obesity preparing for bariatric surgery. The use of a standardized yoga protocol, high-quality bio-banking procedures, and advanced sequencing techniques strengthens the validity of the results, and the longitudinal design and integration with a bariatric-surgery pathway make the findings relevant to clinical practice.

### 5.3 Future directions

- Extend the intervention duration (e.g., 6–12 months) to better match epigenome-remodeling timescales
- Incorporate randomised controlled trial (RCT) designs with allocation concealment and, where feasible, blinded outcome assessment
- Use multi-tissue and multi-omics analyses
- Include larger, multi-center, sociodemographically diverse cohorts to improve generalizability
- Systematically record diet, physical activity, and medication use as covariates
- Explore individual response patterns using stratified or personalized-medicine approaches

## 6 Conclusion

This study provides preliminary evidence that a structured yoga program integrated into the preoperative management of patients with severe obesity is associated with a significant baseline-adjusted difference in plasma noradrenaline and a nominally significant change in circulating TNF-α protein in the unadjusted analysis. The unadjusted SF-36 physical functioning and vitamin D3 findings at T1 were not confirmed by baseline-adjusted ANCOVA; the postoperative vitamin D3 difference was borderline (*p* = 0.064). The noradrenaline difference was observed within a relatively short three-month period and occurred independently of weight loss, highlighting yoga’s potential role as a non-pharmacological adjunct to conventional obesity care.

No significant changes in DNA methylation of the targeted inflammatory genes were detected, though the molecular data generated offer a foundation for more comprehensive epigenetic investigations; the absence of detectable epigenetic modulation may reflect the short intervention duration, peripheral tissue sampling, or the chronic inflammatory state of the study population.

These findings suggest that mind–body practices like yoga may be associated with physiological readiness for surgery, while psychological outcomes should remain interpreted as secondary and exploratory. Larger randomised trials with extended follow-up, systematic confounder control, and multi-omics analysis are warranted to further elucidate the mechanistic underpinnings of yoga’s associations with obesity and metabolic disease and to determine its full potential as a complementary strategy in bariatric care.

## Data Availability

The raw data supporting the conclusions of this article will be made available by the authors, without undue reservation.
